# Combination of Rapamycin and MK-2206 Induced Cell Death *via* Autophagy and Necroptosis in MYCN-Amplified Neuroblastoma Cell Lines

**DOI:** 10.3389/fphar.2020.00031

**Published:** 2020-02-14

**Authors:** Yudi Dong, Wei Gong, Zhongyan Hua, Bo Chen, Guifeng Zhao, Zhihui Liu, Carol J. Thiele, Zhijie Li

**Affiliations:** ^1^Department of Pediatrics, Shengjing Hospital of China Medical University, Shenyang, China; ^2^Medical Research Center, Liaoning Key Laboratory of Research and Application of Animal Models for Environmental and Metabolic Diseases, Shengjing Hospital of China Medical University, Shenyang, China; ^3^Cellular & Molecular Biology Section, Pediatric Oncology Branch, National Cancer Institute, National Institutes of Health, Bethesda, MD, United States

**Keywords:** neuroblastoma, rapamycin, MK-2206, autophagy, necroptosis, MYCN

## Abstract

Neuroblastoma (NB) is the most common pediatric malignant extracranial solid tumor. Despite multi-modality therapies, the emergence of drug resistance is an obstacle in the treatment of high-risk NB patients (with MYCN amplification). In our previous study, we found that rapamycin and MK-2206 synergistically induced cell death in MYCN-amplified cell lines but the mechanisms remained unclear. In our present study, either 3-MA or necroatatin-1 blocked the cell death induced by rapamycin and MK-2206, but z-VAD-fmk did not block this cell death. The expressions of autophagy markers (ATG5, ATG7, Beclin-1, LC3 B) and the necroptosis marker RIPK3 increased and another necroptosis marker RIPK1 decreased after the combination treatment of rapamycin and MK-2206, and were accompanied by the morphological characteristics of autophagy and necroptosis. In NB xenograft tumor tissues, the expressions of autophagy and necroptosis markers were consistent with observations *in vitro*. These data suggested that autophagy and necroptosis contributed to the cell death induced by rapamycin and MK-2206 in NB cells. To understand the role of MYCN in this process, MYCN expression was downregulated in MYCN-amplified cell lines (NGP, BE2) using siRNAs and was upregulated in MYCN non-amplified cell lines (AS, SY5Y) using plasmid. We found the cell death induced by rapamycin and MK-2206 was MYCN-dependent. We also found that the metabolic activity in NB cells was correlated with the expression level of MYCN. This study delineates the role of MYCN in the cell death induced by combination treatment of rapamycin and MK-2206 in MYCN-amplified NB cells.

## Introduction

Neuroblastoma (NB) is the most common pediatric malignant extracranial solid tumor and is derived from cells of the neural crest. Although its incidence is only 7%–8%, it contributes to 15% of all pediatric cancer mortality (Smith et al., 2010; Gatta et al., 2014). The current treatments include chemotherapy, radiotherapy, surgery, biotherapies and immunotherapies. According to the international NB stage system (INSS), patients' clinical stage, histological grade and MYCN gene amplification, NB patients are divided into low, moderate, and high risk groups (Maris et al., 2007). Despite multi-modality therapies, one of the barriers to treatment in high-risk patients with NB is drug resistance. Therefore, novel agents for the NB treatment are urgently needed.

MK-2206, an allosteric AKT inhibitor, binds to the AKT protein in the pleckstrin homology domain resulting in a conformational change of AKT that inhibits the AKT activation by preventing its localization to the plasma membrane (Lindsley et al., 2007). As an anticancer drug, MK-2206 has been proved to be effective both in adult tumors (Djuzenova et al., 2019; Hayasaka et al., 2019) and in a spectrum of pediatric tumors (Gorlick et al., 2012). Phase I studies showed that an oral dose of MK-2206 ranging from 0.25 to 100 mg was well-tolerated (Trucksis et al., 2009). Another larger phase I exploration of MK-2206 is underway to examine dose formulations and combined application with cytotoxic drugs (Hudis et al., 2013). In addition, some studies have shown that MK2206 synergized with molecularly targeted drugs (mTOR inhibitors, MEK inhibitors, cisplatin) in lung cancer, breast cancer, ovarian cancer and tuberous sclerosis complex (Hirai et al., 2010; Meng et al., 2010; Choi et al., 2016; Ji et al., 2017).

Mammalian target of rapamycin (mTOR) is a serine/threonine protein kinase that regulates the proliferation, death, survival and protein synthesis of cancer cells. Rapamycin is a traditional mTOR inhibitor which forms a complex with immunoglobulin FKBP12 (Benjamin et al., 2011). As one type of mTOR inhibitors, rapamycin exerted effects on cancer growth and survival *in vitro* and showed a promising suppression on tumor growth *in vivo* (Grzmil and Hemmings, 2013; Zhao et al., 2019).

Our previous study showed that rapamycin and MK-2206 synergistically inhibited the growth of some NB cell lines, particularly those with MYCN amplification (Li et al., 2012). MYCN is an oncogene and encodes a transcription factor. MYCN amplification has been used to determine NB prognosis and led to poor therapeutic effect and low survival rate in 40% high-risk patients (Cohn and Tweddle, 2004; Pinto et al., 2015). Targeting stability of MYC family member proteins has been extensively investigated in order to develop new pharmacologic strategies against various cancers (Boboila et al., 2018; Wang et al., 2018; Hu et al., 2019). Our previous study showed that the caspase3/7 activity did not significantly increase in the NB cells treated with rapamycin and MK-2206, indicating that NB cell death induced by this combination of rapamycin and MK-2206 was caspase-independent (Li et al., 2012). To investigate the mechanisms of this cell death induced by rapamycin and MK-2206, we performed microarray analysis of BE2 cells treated with rapamycin and MK-2206. We found that genes involved in autophagy and necroptosis were significant enriched. Thus, we investigated the contribution of autophagy and necroptosis to the cell death induced by combination treatment of rapamycin and MK-2206 and evaluated whether this was MYCN-dependent.

## Materials and Methods

### Reagents

Rapamycin and MK-2206 were purchased from Selleckchem (Houston, TX, USA). 3-Methyladenine (3-MA) (M9281) and necrostatin-1 (Nec-1) (N9037) were purchased from Sigma-Aldrich (St. Louis, MO, USA). Pan-caspase inhibitor z-VAD-fmk was purchased from ApexBio (Houston, TX, USA). Primary antibodies anti-LC3 A/B, anti-ATG5, anti-ATG7, anti-Beclin-1, and anti-RIPK3 used for Western Blot were purchased from Cell Signaling Technology (Beverly, Mass, USA), anti-RIPK1 antibody used for Western Blot was purchased from Santa Cruz (Beverly, Mass, USA) and anti-GAPDH antibody was purchased from Kangchen biotech (Shanghai, China). Anti-RIPK1 and anti-RIPK3 antibodies used for immunohistochemistry staining were purchased from Proteintech Group (Rosemont, IL, USA).

### Cell Culture and Treatments

Four human NB cell lines [MYCN-amplified cell lines: NGP and SK-N-BE2 (BE2), MYCN-non amplified cell lines: SH-SY5Y (SY5Y) and SK-N-AS (AS)] were used in our study and were obtained from CT (National Institutes of Health, National Cancer Institute, USA). NB cells were cultured at 37°C with 5% CO_2_ in RPMI-1640 medium (Biological Industries, Israel) containing 10% fetal bovine serum (Biological Industries, Israel), 100 U/ml penicillin, 100 μg/ml streptomycin (Biological Industries, Israel) and 2 mM/L glutamine (Biological Industries, Israel). To assess synergy in NB cells, rapamycin was given 2 h before MK-2206. To study the involvement of autophagy or necroptosis, cells were pretreated with 3-MA or Nec-1 for 2 h before addition of rapamycin and MK-2206.

### Cell Viability Assay

To detect the cell survival, CCK-8 assay (Biotool, Shanghai, China) was used according to the manufacturer's specification. NGP or BE2 cells were seeded in a 96-well plate at the density of 3 × 10^4^/well for 24 h. Cells were treated with rapamycin and MK-2206 for 60 h, or were pretreated with 3-MA, Nec-1 or z-VAD-fmk prior to the addition of rapamycin and MK-2206. Subsequently, CCK-8 was added to each well and incubated for 1 h. Cell viability was then quantified by measuring absorbance at 450 nm optical density. Cell viability was assessed as a percentage of absorbency relative to the control with vehicle treatment as the control. YOYO-1 (Thermo Fisher Scientific, MA, USA) is a high affinitive nucleic acid dye that stains dead cells. IncuCyte Zoom (Essen BioScince, MI, USA) was used to dynamically observe morphology of cells and cell confluence (%) was calculated by phase-contrast images.

### Cell Transfection

Small interfering RNAs (siRNAs) purchased from Ruibo (Guangzhou, China) were used to knock down MYCN. The sequences of siRNAs were:

MYCN-siRNA1: CGGAGTTGGTAAAGAATGA;
MYCN-siRNA2: CGGAGATGCTGCTTGAGAA;
MYCN-siRNA3: CCAAAGGCTAAGAGCTTGA.

NGP and BE2 cells were seeded 2 × 10^5^/ml in 6-well plate. The siRNAs were transfected into cells using jetPRIME (Polyplus Transfection, Illkirsch, France) and after 24 h, cells were treated with rapamycin and MK-2206.

MYCN expression plasmids were isolated using the HiSpeed Plasmid Maxi Kit (Qiagen, Germany) according to the manufacturer's instructions. AS and SY5Y cells (1 × 10^5^/ml) were seeded in 6-well plate and transfected with MYCN plasmids using jetPRIME. After 24 h, cells were treated with rapamycin and MK-2206.

### Transmission Electron Microscope

Cells were treated with rapamycin and MK-2206 for 12 h. Cells were fixed with 2.5% glutaraldehyde overnight at 4°C, washed with 0.1 M natrium cacodylicum for three times and then fixed with 1% osmium tetroxide at 4°C for 90 min. After fixation, the samples were dehydrated in ethyl alcohol and acetone. After the embedding and solidification process, ultrathin sections (60–80 nm) were double stained with 4% uranyl acetate and 0.1% lead citrate, and examined under a transmission electron microscopy (Hitachi, Japan).

### Western Blot

Protein lysates were extracted using RIPA buffer (Beyotime, Shanghai, China) supplemented with PMSF (Beyotime, Shanghai, China). The protein concentration was determined by BCA (Beyotime, Shanghai, China). Total protein in each group was loaded in SDS-PAGE gels and transferred to PVDF membranes (Millipore, Bedford, MA, USA). Membranes were blocked with 5% skim milk (BD, New Jersey, USA) at 4°C overnight and incubated with the anti-LC3A/B antibody (1:1000), anti-ATG5 antibody (1:1000), anti-ATG7 antibody (1:1000), anti-Beclin-1 antibody (1:1000), anti-RIPK3 antibody (1:1000), anti-RIPK1 antibody (1:100), and anti-GAPDH antibody (1:5000) at 4 °C overnight.

### Cell Mitochondrial Stress Assay, Cell Glycolytic Stress Assay and Cell Energy Phenotype Assay

To determine glycolytic rate and mitochondrial stress in NGP and AS cells, an XFe96 Analyzer (Agilent Technologies, USA) was used according to the manufacturer's protocols. Cells were seeded in XFe96 cell culture plates at a concentration of 6,000 NGP cells/well and 3,000 AS cells/well in growth medium. The next day, the cells were treated with the combination of 10 nmol/L rapamycin and 10 μmol/L MK-2206 in fresh medium for 24 h. Prior to the assay (1 h), the culture medium was replaced with Seahorse XF assay medium (pH = 7.4) supplemented with 2 mM glutamine, 1 mM pyruvate and 10 mM glucose and incubated at 37°C for 1 h in a CO_2_ free incubator. Carbonyl cyanide 4-trifluoromethoxyphenylhydrazone (FCCP, a mitochondrial oxidative phosphoric acid dissolution coupling agent), oligomycin (an inhibitor of the ATP synthase subunit F1F0), Glucose, 2-deoxyglucose (2-DG, a glycolysis inhibitor), rotenone and antimycin A (Rot/AA, the respiratory chain complex I and III inhibitors) were used at final concentrations of 1 mM, 1 μM, 1 mM, 0.5 μM, and 0.5 mM, respectively. XFe96 Extracellular Flux Analyzer (Seahorse Bioscience, Billerica, MA, USA) was used to measure the oxygen consumption rate (OCR) and extracellular acidification rate (ECAR) in real-time.

### *In Vivo* Studies

NGP cells were cultured in 10% fetal bovine serum RPMI-1640, harvested and washed with Hank balanced salt solution (HBSS), and re-suspended in HBSS. Cell suspension (100 μl) containing 4 × 10^6^ NGP cells was implanted into the right armpits of BALB/c female nude mice (14–16 g) (Huafukang, Beijing, China). MK-2206 (200 mg/kg) was administered by oral gavage three times a week and rapamycin (5 mg/kg) was given by intraperitoneal injection every day. Rapamycin and MK-2206 were given for 10 d. A section of the tumor tissues was frozen immediately in liquid nitrogen for western blot and another section of the tumor tissues was fixed in 10% formalin for the HE stand immunohistochemistry staining. All experiments involving animals followed the guidelines of Animal Center of Shengjing Hospital of China Medical University, and all animal experimental protocols were approved by the experimental Animal Ethical Committee of Shengjing Hospital of China Medical University (2014PS48K).

### Hematoxylin and Eosin (HE) Staining

NGP tumor tissues were fixed in 4% paraformaldehyde for 24 h, dehydrated with graded ethanol and transparentized with xylene. After the tumor tissues were embedded with paraffin, they were sectioned into 3 μm thickness slices. After staining with hematoxylin and eosin, the slices were observed under a Nikon 300 microscope.

### Immunohistochemistry Staining

Paraffin sections were dewaxed with xylene and were dehydrated with graded ethanol in turn and sectioned into 3 μm thickness slices. Next, slices were retrieved by citric acid buffer (PH = 6.0) microwave antigen retrieval and cooled to room temperature. The slices were treated with 3% H_2_O_2_ for endogenous peroxidase inactivation and goat serum albumin to block nonspecific binding site. Then sections were incubated with anti-RIPK1 (1:50), anti-RIPK3 (1:50) overnight at 4°C and incubated with goat anti-rabbit IgG-HRP for 30 min at room temperature. Finally, diaminobenzidine (DAB) was used to visualize the immunocomplexes under a Nikon 300 microscope.

### Statistical Analysis

Data were expressed as mean ± SD. Statistical analysis were carried out with GraphPad Prism 6. Comparisons between 2 groups were carried out with Student's t-test. P < 0.05 was considered statistically significant.

## Results

### Cell Death Induced by the Combination of Rapamycin and MK-2206 Was Caspase-Independent in NB Cells

**Figure 1:**
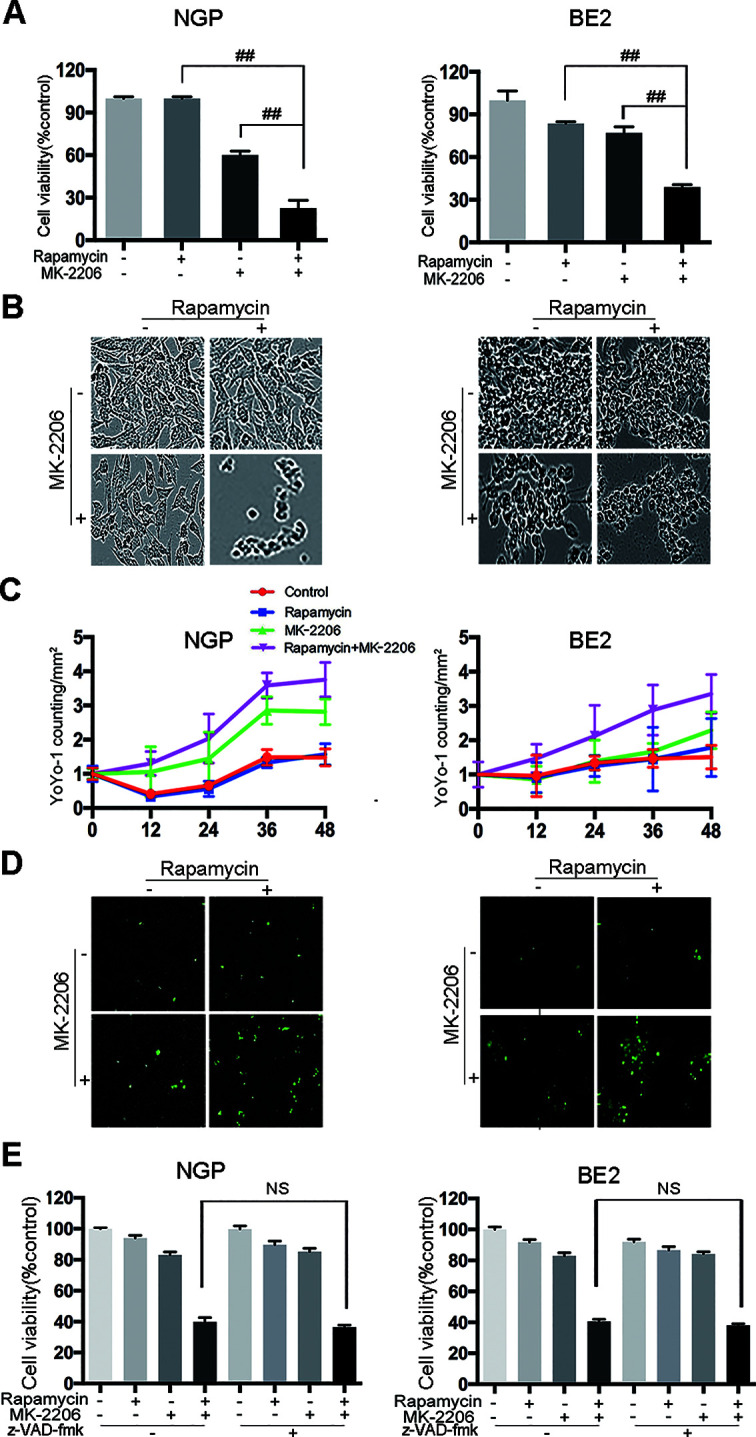
Cell death is induced by the combination of rapamycin and MK-2206 in neuroblastoma (NB) cells. NGP and BE2 cells were treated with rapamycin (10 nmol/L) for 2 h followed by MK-2206 (10 μmol/L) treatment for 48 h. **(A)** Cell viability was evaluated by CCK-8 assay. Bar, SD. ^##^, P < 0.01 (rapamycin+MK-2206 group VS rapamycin or MK-2206 group). **(B)** The live cell morphology was photographed using IncuCyte Zoom. **(C)** Cells were stained with YOYO-1 (1:10000) and dead cells were evaluated by YOYO-1 counting. **(D)** The dead cell morphology stained by YOYO-1 was photographed using IncuCyte Zoom. **(E)** NGP and BE2 cells were pretreatment with pan-caspase inhibitor z-VAD-fmk (100 μM) for 2 h followed by rapamycin (10 nmol/L) and MK-2206 (10 μmol/L) treatment for 48 h, either alone or in combination. Cell viability was evaluated by CCK-8 assay. Bar, SD. All experiments were conducted for three times. NS, no statistical significance.

To investigate the effect of rapamycin and MK-2206 on the cell viability in MYCN-amplified NGP and BE2 cells, we performed CCK-8 assay and IncuCyte Zoom cell confluence assays. The CCK8 assay results showed that the combination of rapamycin and MK-2206 led to a significant decrease of cell viability compared to the control or either drug alone after 48 h of treatment (**Figure 1A**), which was consistent with our previously report (Li et al., 2012). The cell morphology changes after drug treatment were shown in **Figure 1B**. YOYO-1 stained dead cells and YOYO-1 counting showed that the number of dead cells in rapamycin+MK-2206 group was significantly higher than those in the control group or either drug alone after 48 h treatment (**Figures 1C, D**), which was consistent with the CCK-8 assay result (**Figure 1A**). To investigate whether the cell death induced by rapamycin and MK-2206 was caspase-dependent, NGP and BE2 cells were pretreated with pan-caspase inhibitor z-VAD-fmk before administration of rapamycin and/or MK-2206. Pretreatment of the NB cells with z-VAD-fmk before the administration of rapamycin+MK-2206 resulted in a 5.5% difference in NGP cells and 2.5% difference in BE2 cells for the cell viability, indicating that z-VAD-fmk did not block the cell death induced by a combination of rapamycin and MK-2206 (**Figure 1E**). These data indicated that the combined application of rapamycin and MK-2206 induced more cell death than either drug alone in NGP and BE2 cell lines and the cell death induced by rapamycin and MK-2206 was caspase-independent.

### Autophagy Contributed to the Cell Death Induced by the Combination of Rapamycin and MK-2206

**Figure 2:**
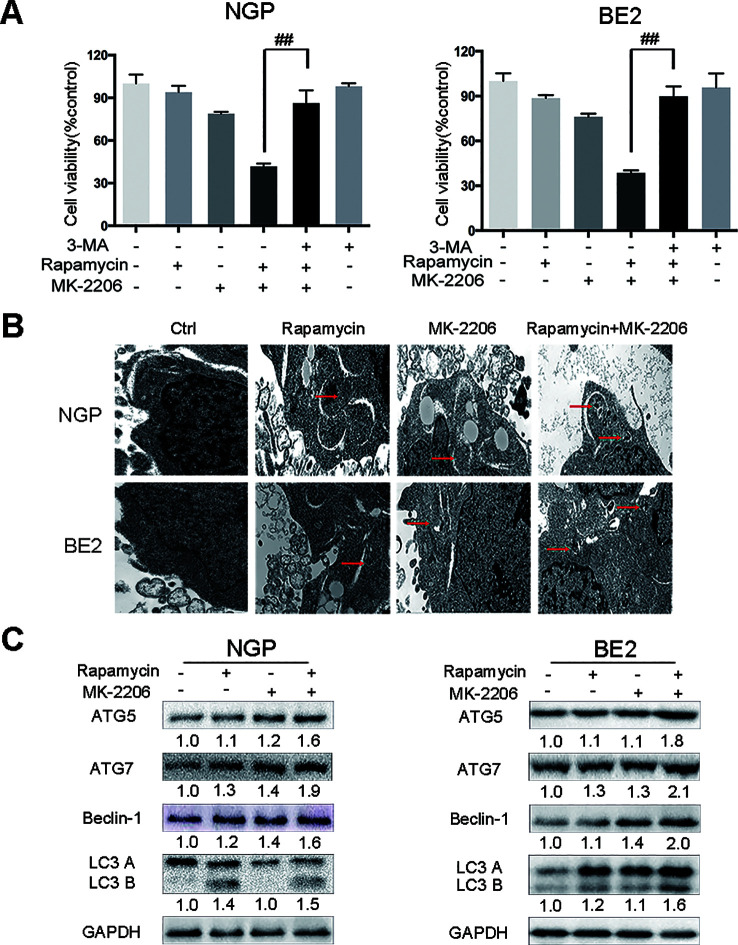
Autophagy mediates the cell death induced by the combination of rapamycin and MK-2206. **(A)** NGP and BE2 cells were pretreated with autophagy inhibitor 3-MA (5 mM) for 2 h followed by rapamycin (10 nmol/L) and MK-2206 (10 μmol/L) treatment, either alone or in combination for 48 h. Cell viability was evaluated by CCK-8 assay. Bar, SD. ^##^, P < 0.01 (3-MA+rapamycin+MK-2206 group vs rapamycin+MK-2206 group). **(B)** The ultrastructural features of NGP and BE2 cells treated with rapamycin, MK-2206 and rapamycin+MK-2206 for 8 h under electron microscopy. **(C)** NGP and BE2 cells were treated with rapamycin (10 nmol/L) for 2 h followed by MK-2206 (10 μmol/L) treatment for 8 h, either alone or in combination. Total protein was extracted to detect ATG5, ATG7, Beclin-1, LC3 B, and GAPDH levels. All experiments were conducted for three times.

To investigate the contribution of autophagy to the cell death induced by the combination of rapamycin and MK-2206, we pretreated NGP and BE2 cells with the autophagy inhibitor 3-Methyladenine (3-MA) prior to rapamycin and MK-2206 treatment. In NGP cells, cell viability was 41.7% in the rapamycin+MK-2206 group while pretreatment with 3-MA significantly increased the cell viability to 86.3% (P < 0.01, rapamycin+MK-2206 group vs rapamycin+MK-2206+3-MA group) (**Figure 2A**, left). In BE2 cells, cell viability was 38.6% in the rapamycin+MK-2206 group but pretreatment with 3-MA significantly increased viability to 89.8% (P < 0.01, rapamycin+MK-2206 group vs rapamycin+MK-2206+3-MA group) (**Figure 2A**, right). We evaluated the changes of cell ultrastructure by transmission electron microscopy in rapamycin+MK-2206 treated NGP and BE2 cells. As shown in **Figure 2B**, compared to the control group, NGP and BE2 cells displayed autophagic vacuoles (AVs) in rapamycin, MK-2206 and rapamycin+MK-2206 treated group; and there were more AVs in the cells treated with the combination of rapamycin and MK-2206 than in the cells treated with each agent alone. We further evaluated the protein expressions of autophagy markers (ATG5, ATG7, Beclin-1, and LC3 B) in NGP and BE2 cells after combination treatment of rapamycin and MK-2206 by Western Blot. Compared to the control or either drug alone, the combination of rapamycin and MK-2206 induced a greater increase in the expression levels of ATG5, ATG7, Beclin-1, and LC3 B in NGP and BE2 cells (**Figure 2C**). These data indicated that autophagy contributed to the cell death induced by the combination of rapamycin and MK-2206.

### Combination of Rapamycin and MK-2206 Induced Cell Death Partially Through Necroptosis

**Figure 3:**
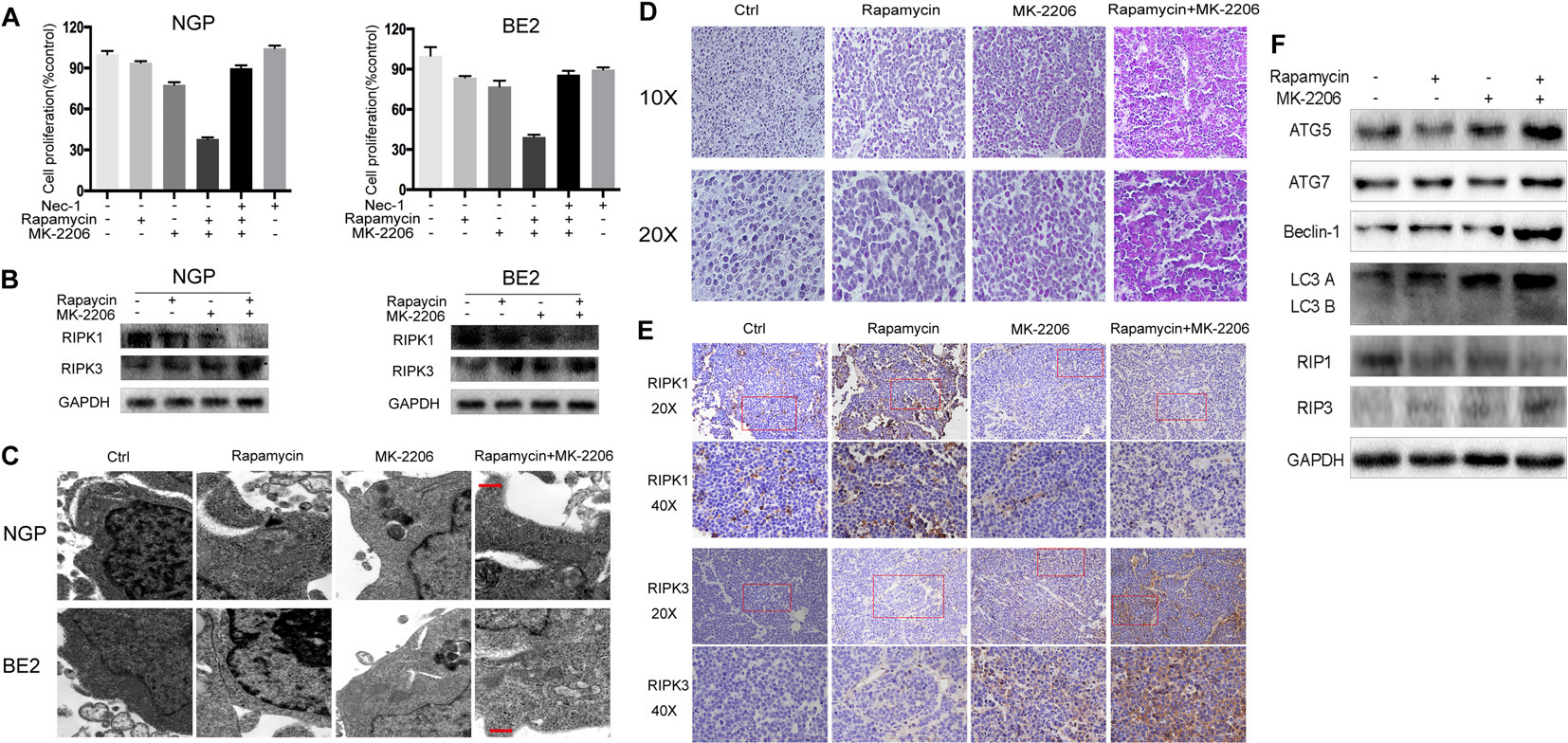
Combination of rapamycin and MK-2206 induces cell death *via* necroptosis. **(A)** NGP and BE2 cells were pretreated with necroptosis inhibitor Nec-1 (40 μM) for 2 h followed by rapamycin (10 nmol/L) and MK-2206 (10 μmol/L) treatment, either alone or in combination for 48 h. Cell viability was evaluated by CCK-8 assay. Bar, SD. ^##^, P < 0.01, (Nec-1+rapamycin+MK-2206 group vs rapamycin+MK-2206 group). **(B)** NGP and BE2 cells were treated with rapamycin (10 nmol/L) for 2 h followed by MK-2206 (10 μmol/L) treatment for 8 h, either alone or in combination. Total protein was extracted to detect RIPK1, RIPK3 and GAPDH levels. **(C)** The ultrastructural features of NGP and BE2 cells treated with rapamycin, MK-2206 and rapamycin+MK-2206 for 8 h under electron microscopy. **(D**–**F)** BALB/c nude mice borne NGP tumors were treated with 5 mg/kg rapamycin and 200 mg/kg MK-2206 for 10 days, either alone or in combination. Tumor tissues were harvested. The morphological changes were observed under microscope after HE staining **(D)**. The expressions of RIPK1 and RIPK3 were detected by immunohistochemistry staining **(E)**. The expressions of autophagy related 5 (ATG5), autophagy related 7 (ATG7), Beclin-1, microtubule associated protein 1 light chain 3 B (LC3 B), receptor interacting serine/threonine kinase 1 (RIPK1), receptor interacting serine/threonine kinase 3 (RIPK3), and GAPDH were detected by Western Blot **(F)**. All experiments were conducted for three times.

To investigate the contribution of necroptosis to the cell death induced by the combination of rapamycin and MK-2206, we pretreated the cells with the necroptosis inhibitor Nec-1. As shown in Fig.3A left panel, NGP cell viability in the rapamycin+MK-2206 group was 38.0%, but in the Nec-1+rapamycin+MK-2206 group cell viability increased to 89.9%. In **Figure 3A** right panel, BE2 cell viability in the rapamycin+MK-2206 group was 39.4%, but the addition of Nec-1 to this combination increased viability to 85.9%, indicating that pretreatment of the NB cells with Nec-1 significantly attenuated the cell death induced by the combination of rapamycin and MK-2206. Western blot results showed that the rapamycin+MK-2206 treatment led to a decrease in the protein level of the necroptosis marker RIPK1 and an increase of necroptosis marker RIPK3 compared to control or single agent treatment in NGP and BE2 cells (**Figure 3B**). Transmission electron microscopy results showed that only the cells treated with the combination of rapamycin and MK-2206 exhibited cytoplasm swelling and plasma membrane integrity deficiency (**Figure 3C**). Therefore, these results indicated that necroptosis contributed to the NB cell death induced by the combination of rapamycin and MK-2206 treatment. We also evaluated the involvement of autophagy and necroptosis in NB xenograft tumor tissues after the combination treatment of rapamycin and MK-2206 in a xenograft murine model. BALB/c nude mice borne NGP tumors were treated with 5 mg/kg rapamycin and 200 mg/kg MK-2206 for 10 d, either alone or in combination. HE staining of the tumor tissues showed that the number of dead cells (with swelled nuclei and lighter staining signal) in rapamycin+MK-2206 group were significantly higher than those in rapamycin or MK-2206 groups (**Figure 3D**). This was similar to the YOYO-1 staining result *in vitro* (**Figure 1D**). To further verify that necroptosis was involved in this cell death, we detected RIPK1 and RIPK3 expression levels in NGP xenograft tumor tissues by immunohistochemistry staining. Compared to the control and single agent group, RIPK1 expression level was downregulated and RIPK3 expression level was upregulated in rapamycin+MK-2206 group (**Figure 3E**). We further evaluated the protein expressions of autophagy markers (ATG5, ATG7, Beclin-1, and LC3 B) and necroptosis markers (RIPK1, RIPK3) in NGP xenograft tumor tissues after rapamycin and MK-2206 combination treatment by Western Blot. Compared to the control and single agent group, the combination of rapamycin and MK-2206 induced more increases of ATG5, Beclin-1, LC3 B, and RIPK3 protein expressions, and induced a decrease in RIPK1 protein expression (**Figure 3F**). These results were consistent with the changes of these markers in NGP cells *in vitro*.

### The Cell Death Induced by the Combination of Rapamycin and MK-2206 Was MYCN-Dependent

**Figure 4:**
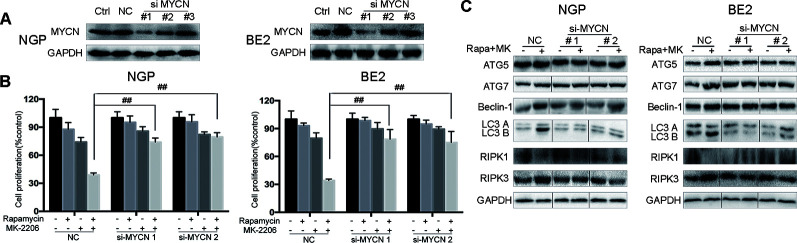
Cell death induced by combination of rapamycin and MK-2206 are attenuated when MYCN is silenced in NGP and BE2 cells. **(A)** Knock down MYCN in NGP and BE2 cells by three siRNA fragments. Total protein was extracted to detect MYCN and GAPDH expressions. **(B)** The NGP and BE2 cells transfected with si-MYCN1 and si-MYCN2 were treated with rapamycin (10 nmol/L) for 2 h followed by MK-2206 (10 μmol/L) treatment for 48 h, either alone or in combination. Bar, SD. ^##^, P < 0.01 (rapamycin+MK-2206 si-MYCN1 group vs rapamycin+MK-2206 NC group and rapamycin+MK-2206 si-MYCN2 group vs rapamycin+MK-2206 NC group). After knocking down MYCN in NGP and BE2 cells by si-MYCN1 and si-MYCN2, cells were treated with rapamycin (10 nmol/L) for 2 h followed by MK-2206 (10 μmol/L) treatment in combination. **(C)** Total protein was extracted to detect autophagy related 5 (ATG5), autophagy related 7 (ATG7), Beclin-1, microtubule associated protein 1 light chain 3 B (LC3 B), receptor interacting serine/threonine kinase 1 (RIPK1), receptor interacting serine/threonine kinase 3 (RIPK3), and GAPDH levels. All experiments were conducted for three times.

**Figure 5:**
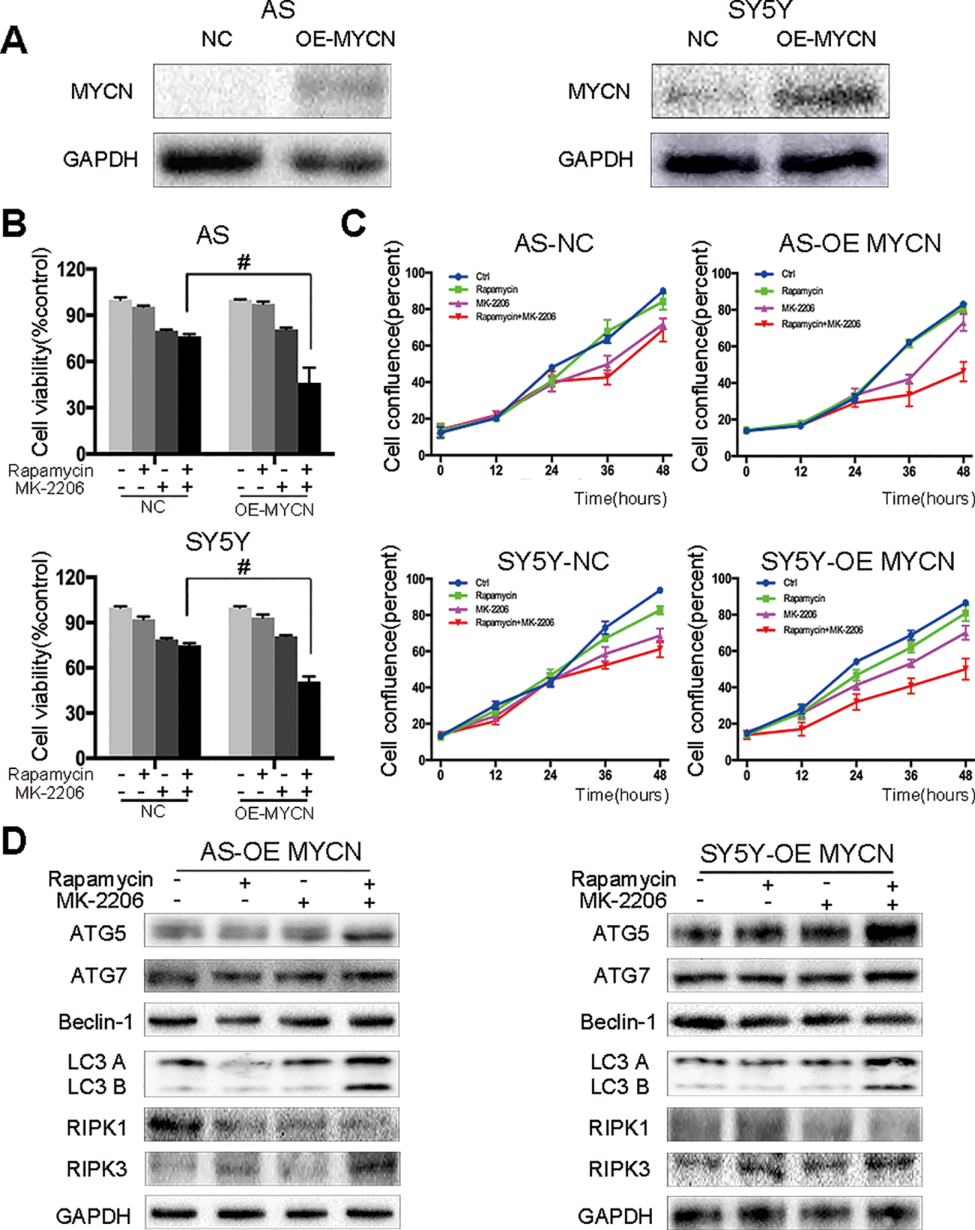
AS and SY5Y cells are more sensitive to the combination of rapamycin and MK-2206 treatment when MYCN is overexpressed. **(A)** Overexpress MYCN in AS and SY5Y cells by plasmid. Total protein was extracted to detect MYCN and GAPDH levels. After overexpressing MYCN in AS and SY5Y cells, cells were treated with rapamycin (10 nmol/L) for 2 h followed by MK-2206 (10 μmol/L) treatment, either alone or in combination. **(B)** Cell viability was detected by CCK-8 assay at 48 h time point. ^#^, P < 0.05 (rapamycin+MK-2206 OE-MYCN group vs rapamycin+MK-2206 NC group). **(C)** Cell confluence was detected by IncuCyte Zoom for 48 h. **(D)** Total protein was extracted to detect autophagy related 5 (ATG5), autophagy related 7 (ATG7), Beclin-1, microtubule associated protein 1 light chain 3 B (LC3 B), receptor interacting serine/threonine kinase 1 (RIPK1), receptor interacting serine/threonine kinase 3 (RIPK3), and GAPDH levels. All experiments were conducted for three times.

To investigate whether the cell death induced by the combination of rapamycin and MK-2206 was MYCN-dependent, we knocked down MYCN using siRNAs in MYCN-amplified NGP and BE2 cell lines (**Figure 4**) and overexpressed MYCN in MYCN non-amplified AS and SY5Y cell lines (**Figure 5**). Among the three MYCN siRNAs, MYCN siRNA1 and siRNA2 were more effective than siRNA3 on knocking down MYCN at protein levels in both NGP and BE2 cells (**Figure 4A**), thus, we chose MYCN siRNA1 and siRNA2 for subsequent experiments. After transfecting MYCN siRNAs in NGP and BE2 cells, we treated the cells with either rapamycin or MK-2206 or in combination. In the control group, the viability of the cells treated with the combination of rapamycin and MK-2206 was 38.7% in NGP cells and 34.2% in BE2 cells. While in the MYCN siRNA transfected cells, the viability of the cells treated with the combination of rapamycin and MK-2206 increased to 73.9% (MYCN siRNA1) and 79.4% (MYCN siRNA2) in NGP cells, and 78.6% (MYCN siRNA1) and 74.9% (MYCN siRNA2) in BE2 cells (**Figure 4B**). We also found that downregulation of MYCN expression in NGP and BE2 cells attenuated the changes of autophagy markers and necroptosis markers that were induced by the combination of rapamycin and MK-2206 (**Figure 4C**). These data indicated that down-regulation of MYCN in NGP and BE2 cells attenuated the cell death induced by the combination of rapamycin and MK-2206.

In our previous study we found that there was no synergistic effect of rapamycin and MK-2206 combination in the MYCN non-amplified AS and SY5Y cells (Li et al., 2012). Here, we overexpressed MYCN in AS and SY5Y cells by transfecting a MYCN expression plasmid (**Figure 5A**). After overexpressing MYCN in AS and SY5Y cells, we treated the cells with either rapamycin or MK-2206 or in combination. Cell viability decreased from 76.34% to 46.21% in AS cells and from 74.93% to 50.76% in SY5Y cells (**Figure 5B**). Cell confluence detected by IncuCyte Zoom showed that the growth curve of the MYCN-overexpressed cells treated with rapamycin+MK-2206 was lower than those of the control group, rapamycin group and MK-2206 group (**Figure 5C**). We also found that in the MYCN overexpressed AS and SY5Y cells, there were higher protein levels of ATG5, ATG7, Beclin-1, LC3 B, and RIPK3, and lower expression of RIPK1 in the cells treated with the combination of rapamycin and MK-2206, compared to the cells in the control group and rapamycin or MK-2206-treated group (**Figure 5D**). These data indicated that upregulation of MYCN expression in AS and SY5Y cells increased the sensitivity of these cells to the treatment of rapamycin+MK-2206 and this was accompanied by an increase in autophagy and necroptosis.

### The Effects of MYCN Expression on Glycolysis and Mitochondrial Respiration

To investigate the relationship between MYCN expression levels and the metabolic processes of NB cells, we analyzed the two main energy-producing cellular processes, glycolysis and aerobic oxidation. OCR and ECAR were measured by the XFe96 Extracellular Flux Analyzer in real time. ECAR represented the glycolysis function and OCR represented the mitochondrial aerobic respiration function.

**Figure 6:**
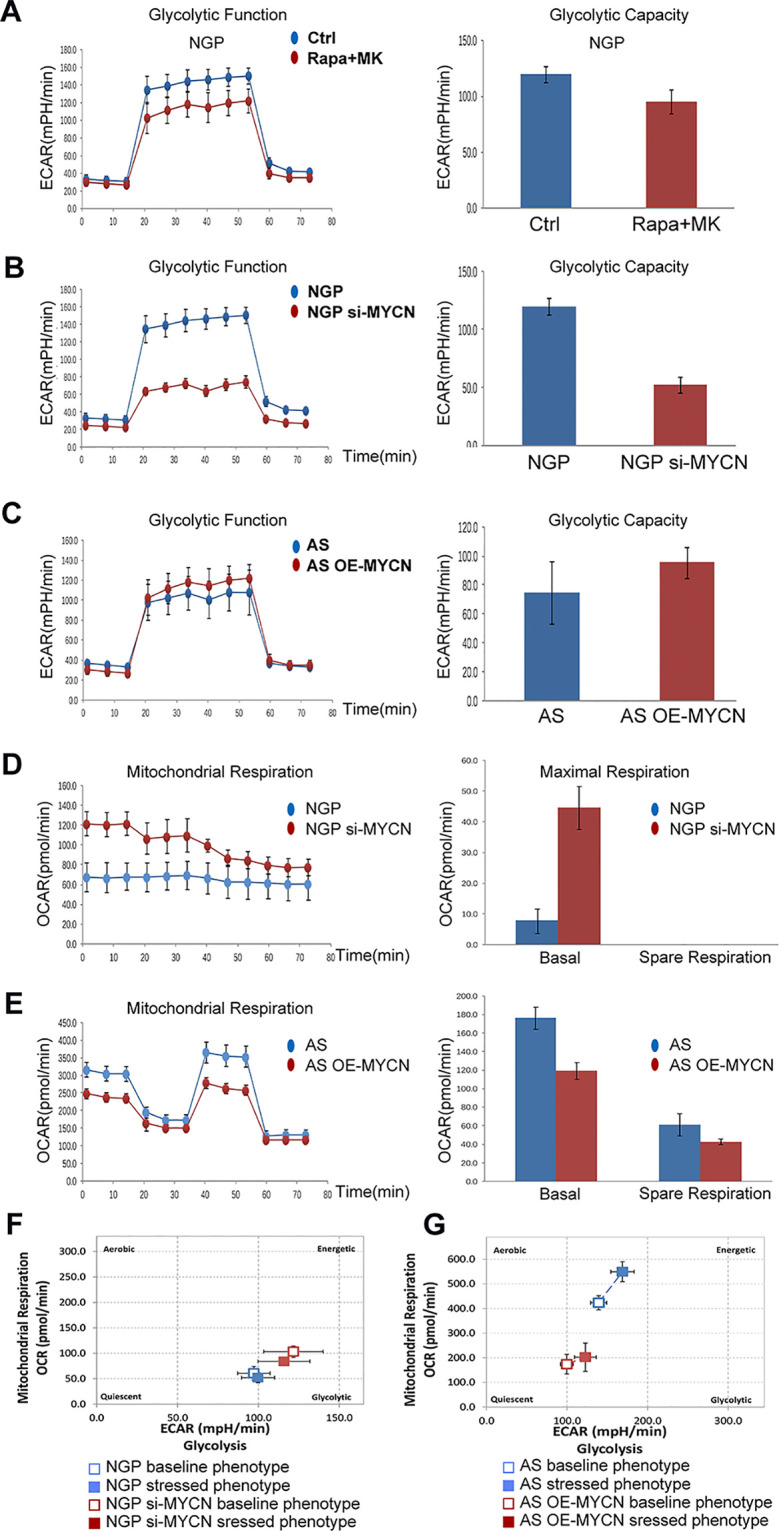
The effects of MYCN expression on glycolysis and mitochondrial respiration. **(A)** The glycolytic function in NGP cells after treatment by combination treatment of rapamycin and MK-2206 for 48 h was assessed and glycolytic capacity was demonstrated by detecting extracellular acidification rate (ECAR. **(B)** The glycolytic function in NGP cells after treatment by knocking down MYCN for 24 h was assessed and glycolytic capacity was demonstrated by detecting ECAR. **(C)** The mitochondrial function in AS cells after treatment by overexpressing MYCN for 24 h was assessed. **(D)** The mitochondrial function in NGP cells after treatment by knocking down MYCN for 24 h was assessed and mitochondrial respiration was demonstrated by detecting OCR. **(E)** The mitochondrial function in AS cells after treatment by overexpressing MYCN for 24 h was assessed. **(F)** The metabolism capacity in NGP cells after treatment by knocking down MYCN for 24 h was assessed. **(G)** The metabolic capacity in AS cells after treatment by overexpressing MYCN for 24 h was assessed. All experiments were conducted for three times.

In NGP cells, after combination treatment of rapamycin and MK-2206, glycolytic capacity decreased in rapamycin+MK-2206 group (**Figure 6A**). To investigate whether the decrease in glycolytic capacity induced by combination of rapamycin and MK-2206 was related to expression of MYCN, we compared the glycolytic capacity (indicated by the changes of ECAR) in NGP and AS cells after the expression of MYCN was modified. There was a significant decrease of ECAR in NGP cells when MYCN was knocked down (**Figure 6B**), while there was an increase in AS cells when MYCN was overexpressed (**Figure 6C**). These data indicated that cells with higher expression of MYCN had higher glycolytic capacity.

We also compared the mitochondrial aerobic respiration function (indicated by the changes of OCR) in NGP and AS cells after the expression of MYCN was modified. There was a significant increase of OCR for the basal respiration in NGP cells when MYCN expression was down-regulated, but a change for the spare respiration capacity was not detected (**Figure 6D**), while there was a decrease for both basal respiration and spare respiration capacity in AS cells when MYCN expression was up-regulated (**Figure 6E**). These data indicated that cells with higher expression of MYCN had lower mitochondrial aerobic respiration function. As shown in **Figure 6F**, after silencing of MYCN expression in NGP cells, the baseline phenotype and the stressed phenotype did not significantly change. In contrast, the baseline phenotype and the stressed phenotype were both increased in MYCN-overexpressed AS cells (**Figure 6G**). These results were the same as the glycolytic and mitochondrial changes in **Figures 6B–E**.

## Discussion

In this study, we found that the combination of rapamycin and MK-2206 treatment induced more cell death in MYCN-amplified NB cells compared to single agent alone, and both autophagy and necroptosis contributed to this cell death in NB cells. The cell death induced by the combination of rapamycin and MK-2206 was MYCN-dependent.

AKT plays an important role in cell signaling and cancer cell growth. mTOR is the downstream of AKT and is present in two distinct complex, mTOR complex 1 (mTORC1) and mTOR complex 2 (mTORC2). mTORC2 is a upstream complex of AKT and promotes cell survival through AKT(Sparks and Guertin, 2010), but for mTORC1, it is a downstream complex of AKT and has a negative feedback effect on AKT in some diseases (Sengupta et al., 2010; Liu et al., 2018). In this case, a combination of AKT inhibitor and mTOR inhibitor may have a better effect compared to each inhibitor alone. Similar to these findings, in our study rapamycin did increase the P-AKT expression in NB cells (data not shown). MK-2206 is an allosteric inhibitor of AKT and has been proved to be effective in inhibiting cancer cell growth. Some other studies showed that combination use of rapamycin and MK-2206 had synergetic effect in multiple myeloma, breast cancer, colon cancer and tuberous sclerosis complex (Ramakrishnan et al., 2012; Ji et al., 2017). MK-2206 had synergetic effects with other mTOR inhibitors like RAD001 in cholangiocarcinoma and hepatocellular carcinoma (Grabinski et al., 2012; Ewald et al., 2013). In NB, Lei Q et al. found that another mTOR inhibitor, AZD8055, prohibited cell growth in MK-2206-resistant NB sublines (Qi et al., 2015). Our previous study showed that rapamycin and MK-2206 had a synergistic effect in some NB cell lines (Li et al., 2012), and we further proved the cell death induced by rapamycin and MK-2206 was caspase-independent in this study. But Neri et al. reported that a combination treatment of MK-2206 with another mTOR inhibitor RAD001 induced a caspase-dependent apoptosis in B-precursor acute lymphoblastic leukemia (B-pre ALL) cells and patient samples. This synergistic effect induced apoptosis through the extrinsic and intrinsic pathways which was accompanied by increases in cleaved caspase-8, caspase-9, and PARP. Furthermore, combination of the two drugs potentiated autophagy induction by increasing LC3 A/B protein level (Neri et al., 2014). However, they did not test whether an autophagy inhibitor could block the cell death induced by RAD001 and MK-2206. Moreover, the mechanism of cell death induced by mTOR inhibitor and MK-2206 in a solid tumor has not been reported.

There are many types of caspase-independent cell death, such as autophagy, necroptosis and pyroptosis (Moreno-Gonzalez et al., 2016; Grootjans et al., 2017). RIPK1 and RIPK3 were both critical regulators of necroptosis. As necroptosis occurred, RIPK3 level consistently increased, but RIPK1 level may increase (Xu et al., 2019; Zhang et al., 2019) or decrease (Motani et al., 2011; Tian et al., 2013) under different stimuli-inducing necroptosis. In our study, the RIPK1 expression decreased after the combination treatment of rapamycin and MK-2206. We detected necroptosis and autophagy markers and used necroptosis and autophagy inhibitors to explore the cell death induced by the combination of rapamycin and MK-2206, and found that necroptosis and autophagy contributed to this cell death.

As an important prognostic marker, the ability to target NB cells expressing MYCN would have potential clinical significance. In Gamble LD's study, inhibition of polyamine uptake with the small-molecule drug AMXT 1501, in combination with ODC1 (ornithine decarboxylase 1) inhibitor DFMO (difluoromethylornithine), inhibited or retarded NB growth and prolonged survival in murine models. Moreover, ODC1 is a direct transcriptional target of MYCN. The authors considered that MYCN was the key to the synergistic effect of the two inhibitors, but the specific mechanism of this action has not been described (Gamble et al., 2019).

Tumor necrosis factor-related apoptosis-inducing ligand (TRAIL) plays an important role in necroptosis. TRAIL sensitivity in NB cells can be improved by reconstituting caspase-8 with IFN-γ and TR2 with chemotherapeutic agents (Yang et al., 2003). MYCN, as a downstream molecule of TRAIL receptors, regulates extracellular death. TRAIL-resistant IMR-32 cells treated with cisplatin resulted in increases in DR5 expression but failed to trigger TRAIL sensitivity. Furthermore, in these cells, the downregulation of MYCN expression and restoration of caspase-8 expression induced apoptosis by activating TRAIL pathway. Pretreatment the cells with cisplatin dramatically enhanced TRAIL cytotoxicity *via* DR5 expression increased (Lee et al., 2019). In our study, combination of rapamycin and MK-2206 produced a synergistic effect in MYCN-amplified cell lines (NGP and BE2), but this synergistic effect was not observed in cell lines without MYCN amplification (AS and SY5Y). Consistently, the knockdown of MYCN in MYCN-amplified NB cells decreased the sensitivity to the combination treatment of rapamycin and MK-2206, while the overexpression of MYCN in MYCN non-amplified NB cells increased the sensitivity to the combination treatment of rapamycin and MK-2206. Despite MYCN amplification predicted poor prognosis and drug resistance, our results indicated that MYCN-amplified NB cells were more sensitive to rapamycin+MK-2206 therapy. Therefore, our study shows the existence of MYCN oncogene addiction in MYCN amplified NB cells, which provides a theoretical basis for the synthetic therapy for MYCN-amplified NB patients.

Glycolysis is a main metabolic mode of tumor cells. The transformation of cancer cells from aerobic respiration to glycolysis plays an important role in the occurrence and development of drug resistance. In drug resistant cells and tissues of breast cancer, gastric cancer, glioblastoma, pancreatic adenocarcinoma and non-small cell lung cancer, glycolytic activity increased and reprogramed, and associated metabolic processes changed had been found (Ruprecht et al., 2017; Ye et al., 2017; Zhao et al., 2017; Liu et al., 2019; Sun et al., 2019). In our study, down-regulation of MYCN in NGP cells resulted in a decrease of glycolysis capacity and an increase of basal and maximal respiration; overexpression of MYCN in AS cells induced the increment of glycolysis capacity and decrement of spare respiration capacity. We also found that the combination treatment of rapamycin and MK-2206 increased the glycolysis of cells and decreased the mitochondrial function through the regulatory effect of MYCN, thus increasing the sensitivity of NB cells to rapamycin+MK-2206. The underlying mechanism remains to be further studied.

In conclusion, our study indicated that autophagy and necroptosis mediated the cell death induced by the combination of rapamycin and MK-2206, and this cell death was MYCN-dependent. A combination use of rapamycin and MK-2206 may be a new strategy for the treatment of high-risk NB patients with MYCN amplification.

## Data Availability Statement

The datasets generated for this study are available on request to the corresponding author.

## Ethics Statement

The animal study was reviewed and approved by the Animal Ethical Committee of Shengjing Hospital of China Medical University.

## Author Contributions

ZLi and YD conceived and designed the study. YD, WG, BC, and GZ performed the study. YD, WG, and ZH analyzed the data. YD and ZLi drafted the manuscript. ZLiu and CT revised the draft of manuscript. All authors corrected and approved the final version of the manuscript.

## Funding

This work was supported by the National Natural Science Foundation of China (Nos. 81472359), Key Research and Development Foundation of Liaoning Province (2019JH8/10300024), 2013 Liaoning Climbing Scholar Foundation and 345 Talent Project of Shengjing Hospital of China Medical University. ZLiu and CT are supported by Center for Cancer Research at the National Institutes of Health in the Intramural Research Program at the NIH.

## Conflict of Interest

The authors declare that the research was conducted in the absence of any commercial or financial relationships that could be construed as a potential conflict of interest.

## Abbreviations

NB, neuroblastoma; ATG5, autophagy related 5; ATG7, autophagy related 7; LC3 A/B, microtubule associated protein 1 light chain 3 A/B; RIPK1, receptor interacting serine/threonine kinase 1; RIPK3, receptor interacting serine/threonine kinase 3; 3-MA, 3-methyladenine; Nec-1, necrostatin-1; HE staining, hematoxylin-eosin staining; siRNA, small interfering RNA; OCR, oxygen consumption rate; ECAR, extracellular acidification rate.

## References

[B1] Benjamin D. Colombi M. Moroni C. Hall M. N. (2011). Rapamycin passes the torch: a new generation of mTOR inhibitors. Nat. Rev. Drug Discovery 10 (11), 868–880. doi: 10.1038/nrd3531 22037041

[B2] Boboila S. Lopez G. Yu J. Banerjee D. Kadenhe-Chiweshe A. Connolly E. P.. (2018). Transcription factor activating protein 4 is synthetically lethal and a master regulator of MYCN-amplified neuroblastoma. Oncogene 37 (40), 5451–5465. doi: 10.1038/s41388-018-0326-9 29880876 PMC6172192

[B3] Choi A. R. Kim J. H. Woo Y. H. Cheon J. H. Kim H. S. Yoon S. (2016). Co-treatment of LY294002 or MK-2206 with AZD5363 Attenuates AZD5363-induced increase in the level of phosphorylated AKT. Anticancer Res. 36 (11), 5849–5858. doi: 10.21873/anticanres.11170 27793908

[B4] Cohn S. L. Tweddle D. A. (2004). MYCN amplification remains prognostically strong 20 years after its “clinical debut”. Eur. J. Cancer 40 (18), 2639–2642. doi: 10.1016/j.ejca.2004.07.025 15571946

[B5] Djuzenova C. S. Fiedler V. Memmel S. Katzer A. Sisario D. Brosch P. K.. (2019). Differential effects of the Akt inhibitor MK-2206 on migration and radiation sensitivity of glioblastoma cells. BMC Cancer 19 (1), 299. doi: 10.1186/s12885-019-5517-4 30943918 PMC6446411

[B6] Ewald F. Grabinski N. Grottke A. Windhorst S. Norz D. Carstensen L.. (2013). Combined targeting of AKT and mTOR using MK-2206 and RAD001 is synergistic in the treatment of cholangiocarcinoma. Int. J. Cancer 133 (9), 2065–2076. doi: 10.1002/ijc.28214 23588885

[B7] Gamble L. D. Purgato S. Murray J. Xiao L. Yu D. M. T. Hanssen K. M.. (2019). Inhibition of polyamine synthesis and uptake reduces tumor progression and prolongs survival in mouse models of neuroblastoma. Sci. Transl. Med. 11 (477), eaau1099. doi: 10.1126/scitranslmed.aau1099 30700572

[B8] Gatta G. Botta L. Rossi S. Aareleid T. Bielska-Lasota M. Clavel J.. (2014). Childhood cancer survival in Europe 1999-2007: results of EUROCARE-5–a population-based study. Lancet Oncol. 15 (1), 35–47. doi: 10.1016/S1470-2045(13)70548-5 24314616

[B9] Gorlick R. Maris J. M. Houghton P. J. Lock R. Carol H. Kurmasheva R. T.. (2012). Testing of the Akt/PKB inhibitor MK-2206 by the pediatric preclinical testing program. Pediatr. Blood Cancer 59 (3), 518–524. doi: 10.1002/pbc.23412 22102563 PMC3290691

[B10] Grabinski N. Ewald F. Hofmann B. T. Staufer K. Schumacher U. Nashan B.. (2012). Combined targeting of AKT and mTOR synergistically inhibits proliferation of hepatocellular carcinoma cells. Mol. Cancer 11, 85. doi: 10.1186/1476-4598-11-85 23167739 PMC3545733

[B11] Grootjans S. Vanden Berghe T. Vandenabeele P. (2017). Initiation and execution mechanisms of necroptosis: an overview. Cell Death Differ. 24 (7), 1184–1195. doi: 10.1038/cdd.2017.65 28498367 PMC5520172

[B12] Grzmil M. Hemmings B. A. (2013). Overcoming resistance to rapalogs in gliomas by combinatory therapies. Biochim. Et Biophys. Acta-Proteins Proteomics 1834 (7), 1371–1380. doi: 10.1016/j.bbapap.2013.01.041 23395884

[B13] Hayasaka N. Takada K. Nakamura H. Arihara Y. Kawano Y. Osuga T.. (2019). Combination of eribulin plus AKT inhibitor evokes synergistic cytotoxicity in soft tissue sarcoma cells. Sci. Rep. 9 (1), 5759. doi: 10.1038/s41598-019-42300-z 30962488 PMC6453888

[B14] Hirai H. Sootome H. Nakatsuru Y. Miyama K. Taguchi S. Tsujioka K.. (2010). MK-2206, an allosteric Akt inhibitor, enhances antitumor efficacy by standard chemotherapeutic agents or molecular targeted drugs *in vitro* and *in vivo*. Mol. Cancer Ther. 9 (7), 1956–1967. doi: 10.1158/1535-7163.MCT-09-1012 20571069

[B15] Hu Q. Zhang B. Chen R. Fu C. A,. J. Fu X.. (2019). ZFHX3 is indispensable for ERbeta to inhibit cell proliferation *via* MYC downregulation in prostate cancer cells. Oncogenesis 8 (4), 28. doi: 10.1038/s41389-019-0138-y 30979864 PMC6461672

[B16] Hudis C. Swanton C. Janjigian Y. Y. Lee R. Sutherland S. Lehman R.. (2013). A phase 1 study evaluating the combination of an allosteric AKT inhibitor (MK-2206) and trastuzumab in patients with HER2-positive solid tumors. Breast Cancer Res. 15 (6), R110. doi: 10.1186/bcr3577 24252402 PMC3979046

[B17] Ji S. Lin W. Wang L. Ni Z. Jin F. Zha X.. (2017). Combined targeting of mTOR and Akt using rapamycin and MK-2206 in the treatment of tuberous sclerosis complex. J. Cancer 8 (4), 555–562. doi: 10.7150/jca.17205 28367235 PMC5370499

[B18] Lee M. W. Kim D. S. Kim H. R. Park H. J. Lee J. W. Sung K. W.. (2019). Inhibition of N-myc expression sensitizes human neuroblastoma IMR-32 cells expressing caspase-8 to TRAIL. Cell Prolif. 52, e12577. doi: 10.1111/cpr.12577 30724400 PMC6536445

[B19] Li Z. Yan S. Attayan N. Ramalingam S. Thiele C. J. (2012). Combination of an allosteric Akt Inhibitor MK-2206 with etoposide or rapamycin enhances the antitumor growth effect in neuroblastoma. Clin. Cancer Res. 18 (13), 3603–3615. doi: 10.1158/1078-0432.CCR-11-3321 22550167 PMC6693338

[B20] Lindsley C. W. Barnett S. F. Yaroschak M. Bilodeau M. T. Layton M. E. (2007). Recent progress in the development of ATP-competitive and allosteric Akt kinase inhibitors. Curr. Top. Med. Chem. 7 (14), 1349–1363. doi: 10.2174/156802607781696864 17692025

[B21] Liu Y. Pejchinovski M. Wang X. Fu X. Castelletti D. Watnick T. J.. (2018). Dual mTOR/PI3K inhibition limits PI3K-dependent pathways activated upon mTOR inhibition in autosomal dominant polycystic kidney disease. Sci. Rep. 8 (1), 5584. doi: 10.1038/s41598-018-22938-x 29615724 PMC5882886

[B22] Liu B. Huang Z. B. Chen X. See Y. X. Chen Z. K. Yao H. K. (2019). Mammalian target of rapamycin 2 (MTOR2) and C-MYC modulate glucosamine-6-phosphate synthesis in glioblastoma (GBM) cells through glutamine: fructose-6-phosphate aminotransferase 1 (GFAT1). Cell Mol. Neurobiol. 39 (3), 415–434. doi: 10.1007/s10571-019-00659-7 30771196 PMC11469801

[B23] Maris J. M. Hogarty M. D. Bagatell R. Cohn S. L. (2007). Neuroblastoma. Lancet 369 (9579), 2106–2120. doi: 10.1016/S0140-6736(07)60983-0 17586306

[B24] Meng J. Dai B. Fang B. Bekele B. N. Bornmann W. G. Sun D.. (2010). Combination treatment with MEK and AKT inhibitors is more effective than each drug alone in human non-small cell lung cancer *in vitro* and *in vivo*. PloS One 5 (11), e14124. doi: 10.1371/journal.pone.0014124 21124782 PMC2993951

[B25] Moreno-Gonzalez G. Vandenabeele P. Krysko D. V. (2016). Necroptosis: a novel cell death modality and its potential relevance for critical care medicine. Am. J. Respir. Crit. Care Med. 194 (4), 415–428. doi: 10.1164/rccm.201510-2106CI 27285640

[B26] Motani K. Kushiyama H. Imamura R. Kinoshita T. Nishiuchi T. Suda T. (2011). Caspase-1 protein induces apoptosis-associated speck-like protein containing a caspase recruitment domain (ASC)-mediated necrosis independently of its catalytic activity. J. Biol. Chem. 286 (39), 33963–33972. doi: 10.1074/jbc.M111.286823 21832064 PMC3190783

[B27] Neri L. M. Cani A. Martelli A. M. Simioni C. Junghanss C. Tabellini G.. (2014). Targeting the PI3K/Akt/mTOR signaling pathway in B-precursor acute lymphoblastic leukemia and its therapeutic potential. Leukemia 28 (4), 739–748. doi: 10.1038/leu.2013.226 23892718

[B28] Pinto N. R. Applebaum M. A. Volchenboum S. L. Matthay K. K. London W. B. Ambros P. F.. (2015). Advances in risk classification and treatment strategies for neuroblastoma. J. Clin. Oncol. 33 (27), 3008–3017. doi: 10.1200/JCO.2014.59.4648 26304901 PMC4567703

[B29] Qi L. Toyoda H. Xu D. Q. Zhou Y. Sakurai N. Amano K.. (2015). PDK1-mTOR signaling pathway inhibitors reduce cell proliferation in MK2206 resistant neuroblastoma cells. Cancer Cell Int. 15, 91. doi: 10.1186/s12935-015-0239-4 26421002 PMC4587771

[B30] Ramakrishnan V. Kimlinger T. Haug J. Painuly U. Wellik L. Halling T.. (2012). Anti-myeloma activity of Akt inhibition is linked to the activation status of PI3K/Akt and MEK/ERK pathway. PloS One 7 (11), e50005. doi: 10.1371/journal.pone.0050005 23185517 PMC3503708

[B31] Ruprecht B. Zaal E. A. Zecha J. Wu W. Berkers C. R. Kuster B.. (2017). Lapatinib resistance in breast cancer cells is accompanied by phosphorylation-mediated reprogramming of glycolysis. Cancer Res. 77 (8), 1842–1853. doi: 10.1158/0008-5472.CAN-16-2976 28209619

[B32] Sengupta S. Peterson T. R. Sabatini D. M. (2010). Regulation of the mTOR complex 1 pathway by nutrients, growth factors, and stress. Mol. Cell 40 (2), 310–322. doi: 10.1016/j.molcel.2010.09.026 20965424 PMC2993060

[B33] Smith M. A. Seibel N. L. Altekruse S. F. Ries L. A. Melbert D. L. O'Leary M.. (2010). Outcomes for children and adolescents with cancer: challenges for the twenty-first century. J. Clin. Oncol. 28 (15), 2625–2634. doi: 10.1200/JCO.2009.27.0421 20404250 PMC2881732

[B34] Sparks C. A. Guertin D. A. (2010). Targeting mTOR: prospects for mTOR complex 2 inhibitors in cancer therapy. Oncogene 29 (26), 3733–3744. doi: 10.1038/onc.2010.139 20418915 PMC3031870

[B35] Sun G. Cheng C. Li X. Wang T. Yang J. Li D. (2019). Metabolic tumor burden on postsurgical PET/CT predicts survival of patients with gastric cancer. Cancer Imaging 19 (1), 18. doi: 10.1186/s40644-019-0205-9 30902116 PMC6431021

[B36] Tian W. Xu D. Han W. He H. Cai H. Chen H.. (2013). Cyclophilin D modulates cell death transition from early apoptosis to programmed necrosis induced by honokiol. Int. J. Oncol. 42 (5), 1654–1663. doi: 10.3892/ijo.2013.1863 23525116

[B37] Trucksis M. Friedman E. Taylor A. Delgado L. Reynders T. DeSmet M.. (2009). A phase I single-rising dose study evaluating the safety, tolerability, pharmacokinetics and pharmacodynamics of an oral akt inhibitor in healthy male volunteers. Cancer Res. 69, 3604.

[B38] Wang Y. Gao S. Wang W. Xia Y. Liang J. (2018). Downregulation of NMyc inhibits neuroblastoma cell growth *via the* Wnt/betacatenin signaling pathway. Mol. Med. Rep. 18 (1), 377–384. doi: 10.3892/mmr.2018.8966 29749516

[B39] Xu Y. Gao H. Hu Y. Fang Y. Qi C. Huang J.. (2019). High glucose-induced apoptosis and necroptosis in podocytes is regulated by UCHL1 *via* RIPK1/RIPK3 pathway. Exp. Cell Res. 382 (2), 111463. doi: 10.1016/j.yexcr.2019.06.008 31247189

[B40] Yang X. Z. Merchant M. S. Romero M. E. Tsokos M. Wexler L. H. Kontny U.. (2003). Induction of caspase 8 by interferon gamma renders some neuroblastoma (NB) cells sensitive to tumor necrosis factor-related apoptosis-inducing ligand (TRAIL) but reveals that a lack of membrane TR1/TR2 also contributes to TRAIL resistance in NB. Cancer Res. 63 (5), 1122–1129. 12615731

[B41] Ye M. Wang S. Wan T. Jiang R. Qiu Y. Pei L.. (2017). Combined inhibitions of glycolysis and AKT/autophagy can overcome resistance to EGFR-targeted therapy of lung cancer. J. Cancer 8 (18), 3774–3784. doi: 10.7150/jca.21035 29151965 PMC5688931

[B42] Zhang Y. Y. Liu W. N. Li Y. Q. Zhang X. J. Yang J. Luo X. J.. (2019). Ligustroflavone reduces necroptosis in rat brain after ischemic stroke through targeting RIPK1/RIPK3/MLKL pathway. Naunyn Schmiedebergs Arch. Pharmacol. 392 (9), 1085–1095. doi: 10.1007/s00210-019-01656-9 31055628

[B43] Zhao H. Duan Q. Zhang Z. Li H. Wu H. Shen Q.. (2017). Up-regulation of glycolysis promotes the stemness and EMT phenotypes in gemcitabine-resistant pancreatic cancer cells. J. Cell Mol. Med. 21 (9), 2055–2067. doi: 10.1111/jcmm.13126 28244691 PMC5571518

[B44] Zhao K. Li X. Chen X. Zhu Q. Yin F. Ruan Q.. (2019). Inhibition of miR-140-3p or miR-155-5p by antagomir treatment sensitize chordoma cells to chemotherapy drug treatment by increasing PTEN expression. Eur. J. Pharmacol. 854, 298–306. doi: 10.1016/j.ejphar.2019.03.034 30980798

