# Corrigendum: Combination of rapamycin and MK-2206 induced cell death via autophagy and necroptosis in MYCN-amplified neuroblastoma cell lines

**DOI:** 10.3389/fphar.2024.1425039

**Published:** 2024-10-16

**Authors:** Yudi Dong, Wei Gong, Zhongyan Hua, Bo Chen, Guifeng Zhao, Zhihui Liu, Carol J. Thiele, Zhijie Li

**Affiliations:** ^1^ Department of Pediatrics, Shengjing Hospital of China Medical University, Shenyang, China; ^2^ Medical Research Center, Liaoning Key Laboratory of Research and Application of Animal Models for Environmental and Metabolic Diseases, Shengjing Hospital of China Medical University, Shenyang, China; ^3^ Cellular & Molecular Biology Section, Pediatric Oncology Branch, National Cancer Institute, National Institutes of Health, Bethesda, MD, United States

**Keywords:** neuroblastoma, rapamycin, MK-2206, autophagy, necroptosis, MYCN

In the published article, there was an error in Figure 3 as published. The Western Blot strip of RIPK1 in NGP cells were duplicated to the RIPK1 strip in BE2 cells in Figure 3B. In the original submission of the manuscript, the correct RIPK1 strip in BE2 cells was pasted in Figure 3B; but in the revised submission of the manuscript, when the layout of Figure 3 was adjusted to fit the revised data, the RIPK1 strip of NGP cells was wrongly duplicated to that of BE2 cells**.** The corrected Figure 3 and its caption appear below.

**FIGURE 3:**
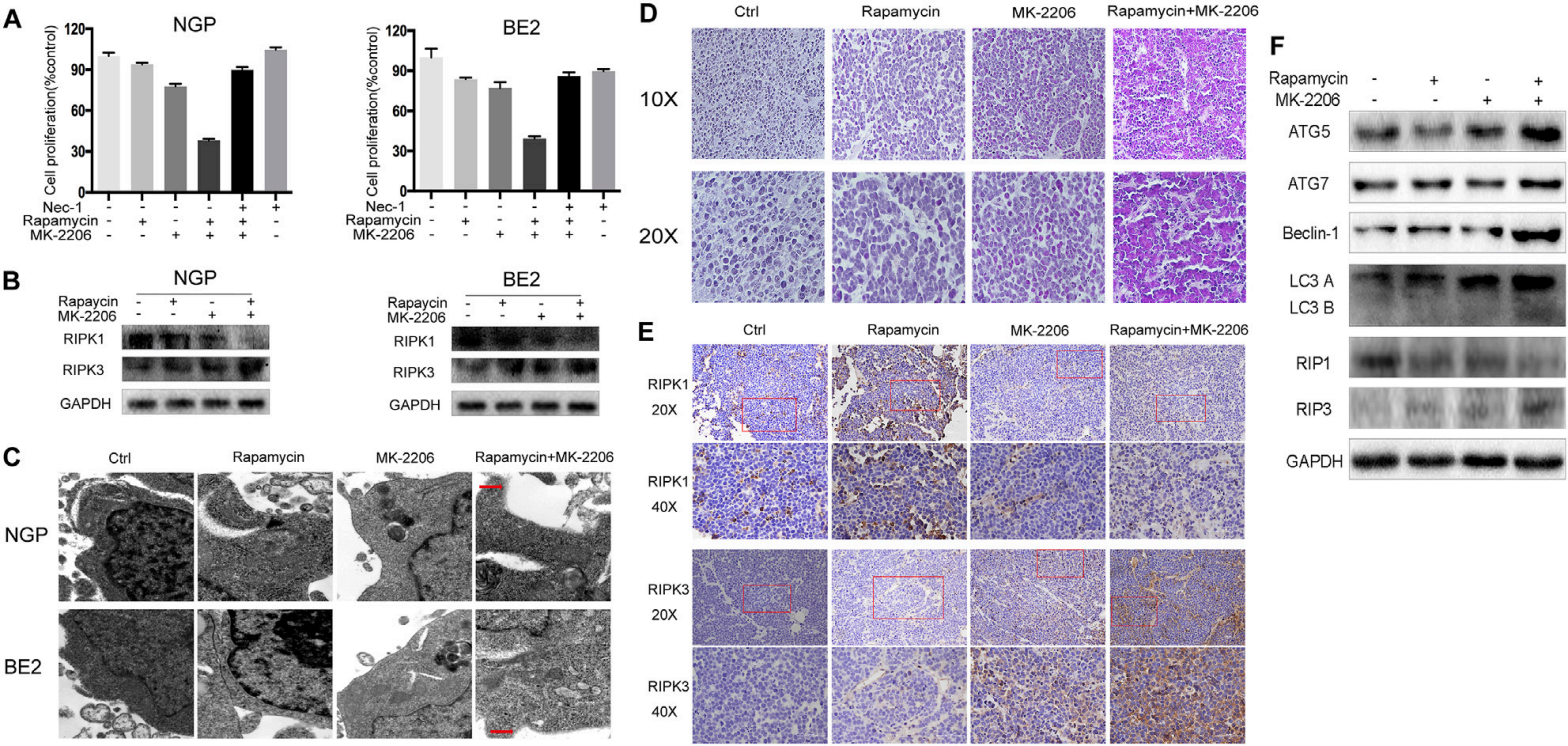
Combination of rapamycin and MK-2206 induces cell death via necroptosis. **(A)** NGP and BE2 cells were pretreated with necroptosis inhibitor Nec-1 (40 mM) for 2 h followed by rapamycin (10 nmol/L) and MK-2206 (10 mmol/L) treatment, either alone or in combination for 48 h. Cell viability was evaluated by CCK-8 assay. Bar, SD. ##, *p* < 0.01, (Nec-1+rapamycin + MK-2206 group vs. rapamycin + MK-2206 group). **(B)** NGP and BE2 cells were treated with rapamycin (10 nmol/L) for 2 h followed by MK-2206 (10 mmol/L) treatment for 8 h, either alone or in combination. Total protein was extracted to detect RIPK1, RIPK3 and GAPDH levels. **(C)** The ultrastructural features of NGP and BE2 cells treated with rapamycin, MK-2206 and rapamycin + MK-2206 for 8 h under electron microscopy. **(D–F)** BALB/c nude mice borne NGP tumors were treated with 5 mg/kg rapamycin and 200 mg/kg MK-2206 for 10 days, either alone or in combination. Tumor tissues were harvested. The morphological changes were observed under microscope after HE staining **(D)**. The expressions of RIPK1 and RIPK3 were detected by immunohistochemistry staining **(E)**. The expressions of autophagy related 5 (ATG5), autophagy related 7 (ATG7), Beclin-1, microtubule associated protein 1 light chain 3 B (LC3 **(B)**, receptor interacting serine/threonine kinase 1 (RIPK1), receptor interacting serine/threonine kinase 3 (RIPK3), and GAPDH were detected by Western blot **(F)**. All experiments were conducted for three times.

The authors apologize for this error and state that this does not change the scientific conclusions of the article in any way. The original article has been updated.

